# Analysis of Light Transport Features in Stone Fruits Using Monte Carlo Simulation

**DOI:** 10.1371/journal.pone.0140582

**Published:** 2015-10-15

**Authors:** Chizhu Ding, Shuning Shi, Jianjun Chen, Wei Wei, Zuojun Tan

**Affiliations:** 1 College of Science, Huazhong Agricultural University, Wuhan, Hubei, China; 2 Institute of Applied Physics, Huazhong Agricultural University, Wuhan, China; Tsinghua University, CHINA

## Abstract

The propagation of light in stone fruit tissue was modeled using the Monte Carlo (MC) method. Peaches were used as the representative model of stone fruits. The effects of the fruit core and the skin on light transport features in the peaches were assessed. It is suggested that the skin, flesh and core should be separately considered with different parameters to accurately simulate light propagation in intact stone fruit. The detection efficiency was evaluated by the percentage of effective photons and the detection sensitivity of the flesh tissue. The fruit skin decreases the detection efficiency, especially in the region close to the incident point. The choices of the source-detector distance, detection angle and source intensity were discussed. Accurate MC simulations may result in better insight into light propagation in stone fruit and aid in achieving the optimal fruit quality inspection without extensive experimental measurements.

## Introduction

Fruit quality inspection techniques play a very important role in the production and consumption of fruits. As visible-near infrared (vis-NIR) spectroscopy measurements can be carried out in a non-destructive way, their potential for fruit grading has been investigated by many research groups worldwide [1]. Unfortunately, the light-based techniques of fruit quality measurement are challenging, and their accuracy and robustness are often limited. Light interaction with fruit tissue is a complicated phenomenon involving both absorption and scattering. While absorption in the vis-NIR range is related to some important chemical quality attributes, such as the total sugar content, scattering is related to the physical properties of the fruit (e.g., density, particle size, and microstructure) [2]. With a knowledge of optical properties of fruit tissues, we can quantitatively understand light transport features inside intact fruit and develop a light source-detector configuration to effectively assess fruit quality. Therefore, there is a growing demand for accurate and fast models to theoretically predict the light distribution in fruits that have given optical properties and to inversely deduce the optical properties from the measurable quantities. The radiation transport equation (RTE) is generally used to describe the light transport in a turbid media.

Although the RTE forms the basis for the general solution of light propagation in absorption/scattering media, it is very difficult to obtain an analytical solution to RTE in complex geometries. The Monte Carlo (MC) simulation method is widely used to ray-trace individual photons in chemicals and complex materials, such in fruits as well as in human tissue [3–7]. Several studies [8–10] have used the MC simulation to investigate light propagation in fruit tissues. However, all of these methods based on the diffusion theory consider the fruit to be a homogeneous semi-infinite turbid media. Actually, most fruits are not homogeneous because they have a surface layer (skin, typically a thick skin). The stone fruit (or drupe) is an indehiscent fruit in which an outer fleshy part surrounds a shell of hardened endocarp with a seed inside. The properties of the surface, the sub-surface layer and the core would inevitably affect the measurement results. Conversely, the optical properties of fruit may depend on many factors, such as the season, orchard, cultivation method, harvest, ripening process, and storage time, even within one cultivar. Because of the large property variations, it is nearly impossible to find fruit samples with only-one parameter difference. Therefore, it is difficult to experimentally analyze the interaction between the fruit flesh, the skin and the core.

Considering that stone fruit is complex and the fruit flesh is quite different from the fruit skin and the fruit core, we intend to develop a MC model of light transport for a heterogeneous fruit tissue and investigate the effects of the fruit core on the light transport features in intact stone fruit. In this paper, peaches were used as the representative model for the stone fruit partly because peach is a soft juicy fruit on the tree and has yellow flesh, dark yellow or red skin, and a stone with a single seed. The migration of a Gaussian beam at a wavelength of 808 nm in an intact peach fruit tissue was simulated using the MC method. The effects of the fruit core and the skin on the major features of the light transport were analyzed. The analysis resulted in better insight into light propagation in stone fruit, which is useful for developing optical techniques for non-destructive fruit quality inspection.

## Materials and Methods

### Estimation of optical properties of peaches

The peach sample set consisted of 10 ‘Mitao’ peaches that were purchased from a local supermarket. These peaches were manually chosen to be as uniform as possible. Experiments were performed after the peaches had been kept at room temperature (~25°C) for at least one day. The spatially resolved diffuse reflectance was measured using intact peaches. Then, on each of the peaches, sets of peach tissues (including the skin and flesh) were cut from the equatorial area. To prepare for the skin samples, all flesh cells were scraped away using a razor blade. All the skin samples and the flesh samples were cut to square pieces. The thickness of each sample was measured using a micrometer. The sample was placed between two 1-mm thick glass sliders. The total diffuse reflectance, the total transmittance, and the collimated transmittance of the peach skin and flesh samples were measured using an integrating sphere system at 808 nm. The absorption coefficient (*μ*_*a*_), the scattering coefficient (*μ*_*s*_), and the anisotropy coefficient (*g*) of peach skin and flesh were calculated using the inverse adding-doubling (IAD) method. The measurement procedure followed the instructions given by Scott Prahl [11, 12]. The refractive indices (*n*) of the tissues were measured by an Abbe refractometer.

### Evaluation of the detection efficiency

Although we are most interested in the properties of fruit flesh tissue in the fruit quality inspection, the radiation has to travel through the skin before it reaches the flesh. In both reflectance and interactance mode, some of the detected photons were diffusely reflected by the skin directly without travelling through the flesh tissue. The photons that penetrated into the flesh layer and were scattered by the flesh tissue were regarded as ‘effective photons’. In the present work, we evaluate the detection efficiency using the percentage of effective photons and the detection sensitivity of the flesh tissue.

The percentage of the effective photons *P*_*eff*_ is defined as the proportion of the number of effective photons to the total number of detected photons:

$$P_{eff}=\frac{N_{eff}}{N_{total}}\times 100\%$$ (1)

The detection sensitivity of the flesh tissue *S*_*flesh*_ is defined as the ratio of the optical pathlength in the flesh tissue to the total optical pathlength:

$$S_{flesh}=\frac{l_{flesh}}{l_{total}}$$ (2)

To increase the detection efficiency, improvements to the *P*_*eff*_ and *S*_*flesh*_ must be made.

### Monte Carlo simulation

Several computational techniques exist to calculate photon propagation through a given media. Among the different approaches available, MC-based methods have been successful because of their ability to test the validity of complex analytical algorithms obtained from the RTE [13]. The MC-based methods have been utilized in a large set of possible optical applications to simulate light distribution in tissues [5, 7, 8, 10, 14–16] and to measure the optical properties of tissues [17–19] by covering wavelengths from the visible to the near infrared (NIR) range.

**Fig 1:**
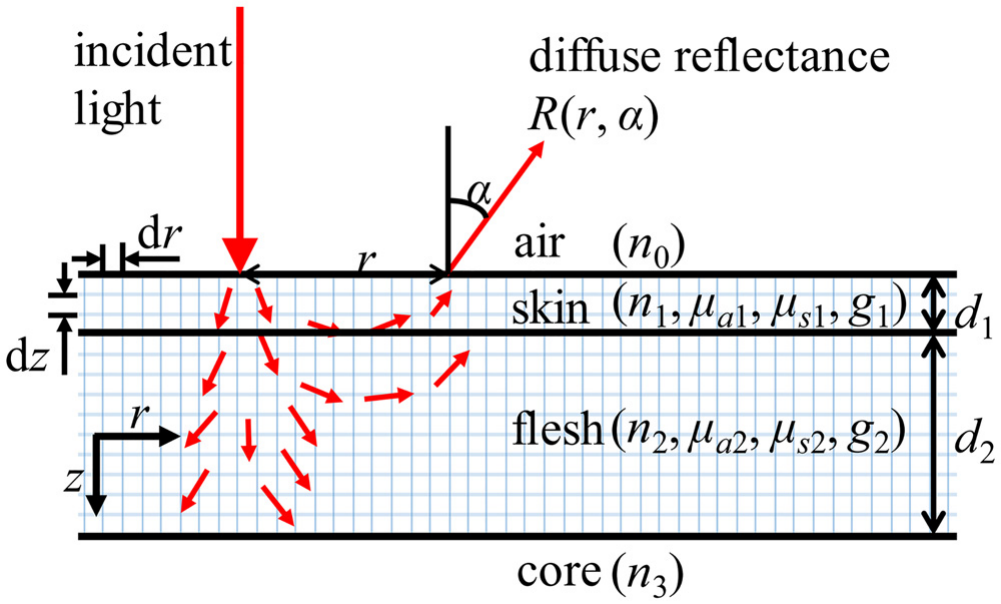
Monte Carlo simulation of light transport in stone fruit.

The MC simulation for light transport in a stone fruit is illustrated in Fig 1. Because the spatial distribution of the photons inside the fruit tissue is largely confined to a small portion of the peach fruit, it was, therefore, reasonable to consider the fruit tissue to be a multi-layered turbid medium consisting of three layers (skin-flesh-core layers). It was further assumed that the skin, flesh and core layers were homogeneous. The surface and interfaces were assumed to be smooth. MC simulation tracks millions of photons from the incident light beam inside the fruit tissue and scores the weights of the scattered and absorbed photons.

#### Photon propagation

When a photon is launched, it is injected orthogonally into the tissue with an initial weight *w*_0_ = 1. If there is a mismatched boundary at the tissue surface, then some specular reflectance will occur, and the photon weight will be decremented by the specular reflectance, *R*_*sp*_. The step size *s* of the photon is determined by the probability distribution of photon’s free path:

$$s=\frac{-\text{ln}(\xi )}{\mu_{a}+\mu_{s}}$$ (3)

where *ξ* is a random number uniformly distributed over the interval (0, 1). When the initial position, the direction and the step size are determined, the photon is ready to be moved in the tissue. During a step, a check must be made to verify if the photon hits the boundary between layers of different optical properties. The coordinates and the propagation direction of the photon are updated, and the photon weight decreases after the move. Then, a new step size is calculated, and the photon moves this new step.

The photon propagation terminates when the photon escapes into the ambient medium or its weight *w* falls below a threshold value (*w*_*th*_ = 10^-4^ in the present work). The roulette technique gives the photon a chance of *m* (e.g., *m* = 10) of surviving with a weight of *mw*. If the photon does not survive the roulette, the photon is terminated, and a new photon is launched and traced thereafter.

#### Photon absorption and scattering

The photon weight is decreased by Eq (4) due to absorption at each interaction.

$$\Delta w=w\frac{\mu_{a}}{\mu_{a}+\mu_{s}}$$ (4)

The photon with the new weight will scatter at the interaction site and change propagation direction. The choice for deflection angle *θ* is determined by the Henyey-Greenstein phase function [20] as a function of the random number *ξ*:

$$\text{cos}\theta =\{\begin{array}{cc} \frac{1}{2g}\left[1+g^{2}-{\left(\frac{1-g^{2}}{1-g+2g\xi }\right)}^{2}\right], & if\;g>0\\ 2\xi -1, & if\;g=0 \end{array}$$ (5)

#### Reflection or transmission

If the photon hits the boundary during a step, then the proportion of internal reflection is defined by Snell’s refraction law and Fresnel’s equations:

$$R(\alpha_{i})=\frac{1}{2}\left[\frac{{\text{sin}}^{2}(\alpha_{i}-\alpha_{t})}{{\text{sin}}^{2}(\alpha_{i}+\alpha_{t})}+\frac{{\text{tan}}^{2}(\alpha_{i}-\alpha_{t})}{{\text{tan}}^{2}(\alpha_{i}+\alpha_{t})}\right]$$ (6)

where *α*_*i*_ and *α*_*t*_ are the incident and refraction angles, respectively. Whether the photon is internally reflected is determined by generating a random number *ξ*, and comparing *ξ* with *R*(*α*_*i*_).

If the photon is internally reflected, then the remaining step size has to been verified again. Conversely, if the photon is refracted into another tissue layer, the remaining step size has to be updated for the new optical properties, and the step size is again checked for boundary crossing. If the photon escapes into the ambient medium, the reflectance *R*(*r*, *α*) at the particular grid element should be incremented by the amount of the escaped photon weight, and the tracing of the propagation of the photon ends here.

#### Other input parameters for the MC simulation

In addition to the optical properties of the skin, flesh and core of the peach fruit model, some other parameters were specified for the MC simulation. These parameters are listed in Table 1. The simulation results obtained by the methods described above are based on the assumption that the light source is an infinitely narrow photon beam. To be more consistent with practical optical systems, the incident light beam was assumed to be a Gaussian light beam with a total energy of 0.5 J and a 1/e^2^ radius of 0.05 cm. The profile of the incident light beam was convolved with the original simulation results to yield the responses to the incident light beam.

**Table 1:** Some input parameters for the MC simulation.

Parameters	Value
Number of photons	3,000,000
Spatial resolution of radial distance (d*r*)	0.01 cm
Spatial resolution of tissue depth (d*z*)	0.01 cm
Number of grids for radial distance *r*	400
Profile of the incident light beam	Gaussian
Total energy of the incident light beam	0.5 J
1/e^2^ radius of the incident light beam	0.05 cm

Given the optical properties of the peach skin, flesh and core, the MC method could generate simulation results for the diffuse reflectance and internal photon absorption with a spatial resolution of 0.01 cm. The publicly available code for MC simulation, which is known as ‘MCML’ and was developed by Wang et al. [13], was modified and applied to simulate light transport in peach fruit. The ‘CONV’ code developed by Wang et al. [3] was used for the convolution for the finite-size incident light beam.

## Results and Discussion

### Experimental validation of the MC simulation

**Fig 2:**
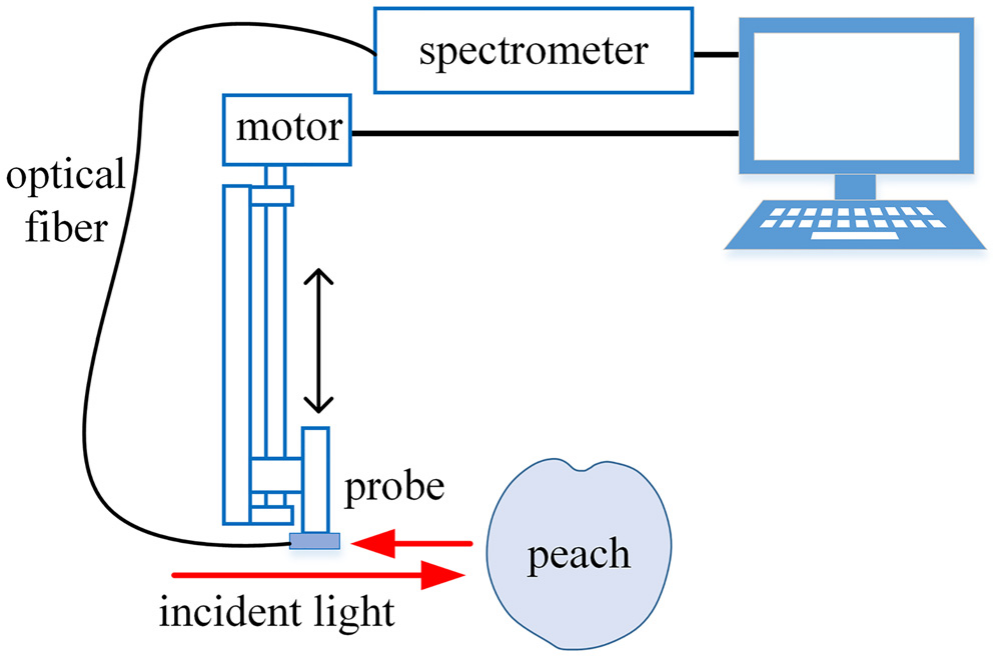
Schematic of the setup for the spatially resolved diffuse reflectance measurements.

The spatially resolved diffuse reflectance measurement system used in this work consisted of a light source, a sensing fiber-optic probe, a spectrometer, a micro-displacement platform, and a computer used for data acquisition and measurement control. The probe was mounted on the micro-displacement platform and collected the diffuse reflected light 0.3 to 2.5 cm away from the incident point. Fig 2 gives a schematic illustration.

**Fig 3:**
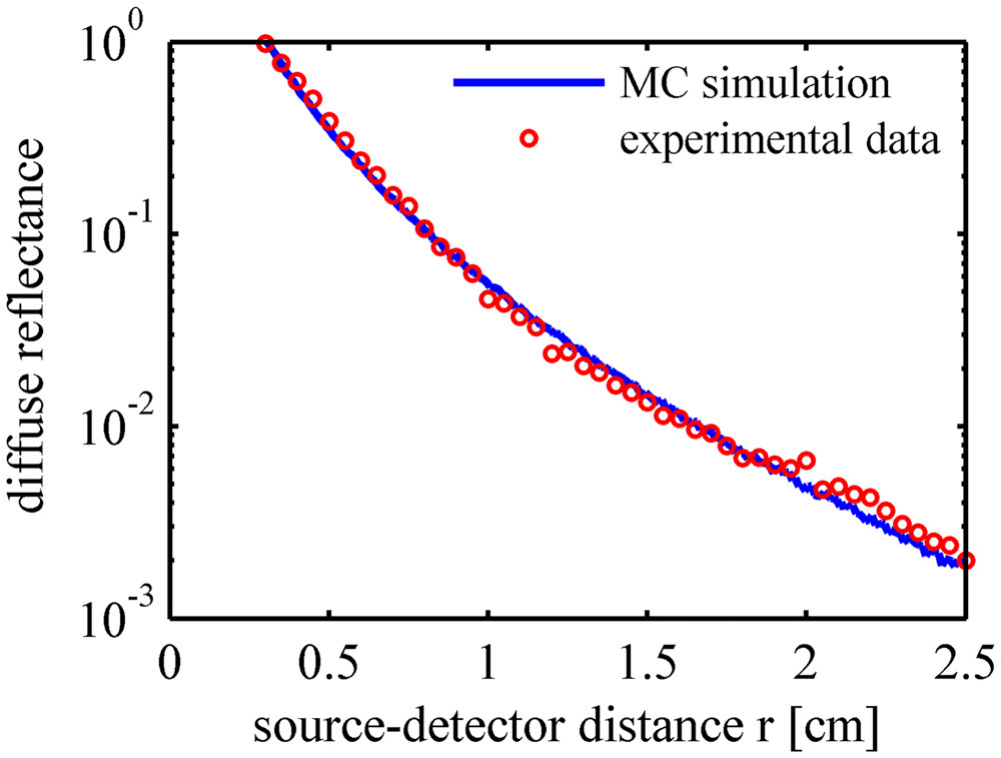
Validation of MC simulation for the spatially resolved diffuse reflectance at 808 nm.

The optical properties of the peaches at 808 nm were used in the MC simulations to validate the simulation results. Although the peach samples were carefully selected to be as uniform as possible, the optical properties of the peaches still varied slightly. The parameters of one selected peach are listed in Table 2. The spatial profiles of the diffuse reflectance from the MC simulation and the experimental data are plotted in Fig 3. The profiles are normalized at the initial point of the experimental data (source-detector distance *r* = 0.3 cm). Fig 3 shows that the simulation results matched the experimental profiles in the source-detector distance ranging from 0.3 to 2.5 cm. The direct comparison result validates the suitability of the MC method.

**Table 2:** Optical properties of one selected peach at 808 nm.

Layer	Parameters	Value
**Medium above (air)**	Refractive index *n*_0_	1.00
**Fruit skin**	Refractive index *n*_1_	1.337
	Absorption coefficient *μ*_*a*1_	0.075 cm^-1^
	Scattering coefficient *μ*_*s*1_	102 cm^-1^
	Anisotropy factor *g*_1_	0.65
	Thickness *d*_1_	0.03 cm
**Fruit flesh**	Refractive index *n*_2_	1.342
	Absorption coefficient *μ*_*a*2_	0.024 cm^-1^
	Scattering coefficient *μ*_*s*2_	28.4 cm^-1^
	Anisotropy factor *g*_2_	0.61
	Thickness *d*_2_	2.34 cm
**Fruit core**	Refractive index *n*_3_	1.46

### Effects of the fruit core

Peach-stone is a single, large seed surrounded by a wood-like husk, which will absorb, scatter and reflect incident light. In the transmittance measurement mode, very high incident light intensity is needed to obtain sufficient light transmission due to the blocking effect of the peach-stone. The light can easily burn the fruit surface and alter its spectral properties due to high power [1]. In both reflectance and interactance mode, the main influence of the stone is that a portion of the incident light will be reflected by the stone and re-absorbed/scattered in the flesh tissue. In the present work, we focused on the reflectance by the stone and simplified it as boundary conditions.

To analyze the effects of the peach-stone on the light transport features in intact peaches, the thickness of the peach skin layer was fixed at *d*_1_ = 0.03 cm. The medium below flesh layer was air with *n*_*below*_ = 1.00 in the simulations of the light propagation in the fruit tissues without cores, whereas *n*_*below*_ = 1.46 was used for the fruit core in the simulations with the peach-stones. The thickness of the peach flesh layer *d*_2_ was changed in the range of 1.5 to 3.0 cm. Other parameters used in the MC simulations were the same with those in Table 2.

The light intensity in the turbid media follows an exponential decrease with the effective attenuation coefficient *μ*_*eff*_ as the decay constant [21].

$$\mu_{eff}=\sqrt{3\mu_{a}(\mu_{a}+{\mu^{\prime }}_{s})}$$ (7)

**Fig 4:**
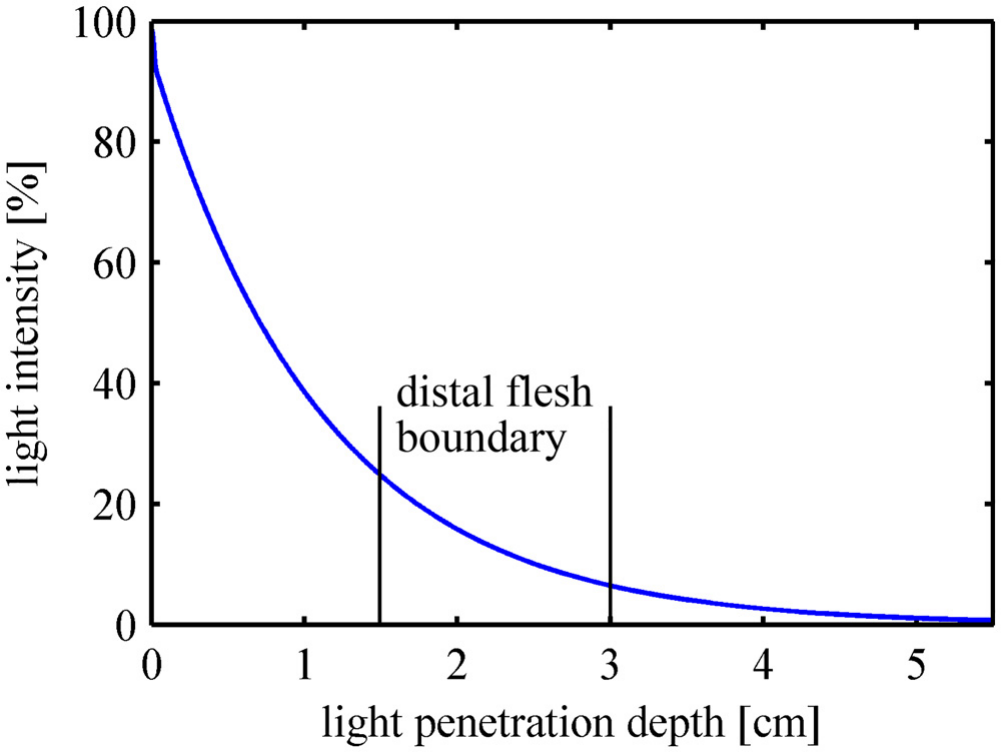
Relationship between light attenuation in the peach tissue and the light penetration depth.

**Fig 5:**
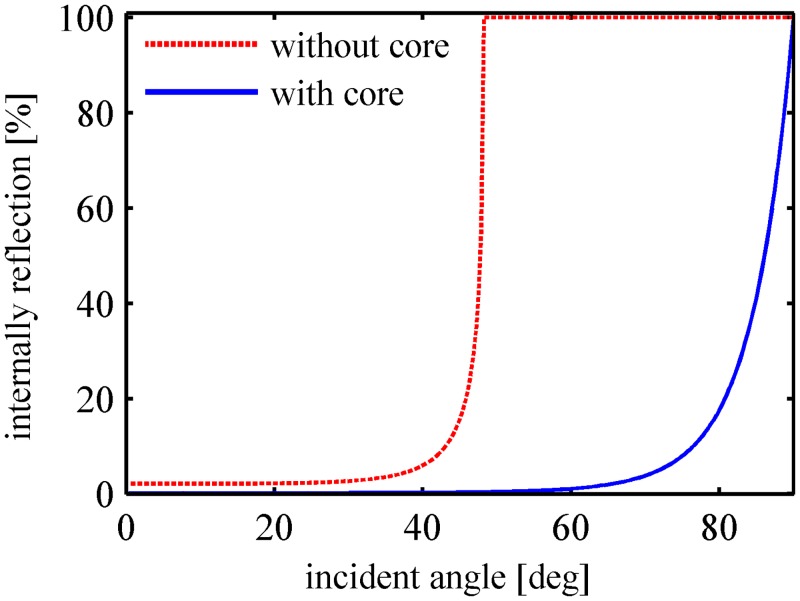
Proportion of the internal reflection at the distal flesh boundary with and without the fruit core.

Fraser *et al*. suggested a 1% penetration depth, which is defined as the distance where the light intensity is reduced to 1% [22]. For the parameters used in our simulation, the relationship between the light attenuation in peach tissue and the light penetration depth is plotted in Fig 4. The 1% penetration depth is 5.08 cm, and approximately 6.4% ~ 24.5% of the light intensity will reach the distal flesh boundary. In Fig 5, the proportion of the internal reflection at the boundary is calculated according to Eq (6). It can be deduced that less light intensity will be internally reflected and re-absorbed/scattered by the flesh tissue if there is a fruit core. The total diffuse reflectance and the fraction of the light absorbed by the flesh tissue will be reduced by the core. A thicker flesh layer results in less influence of the core on light transport features, as less light intensity will reach the distal flesh boundary.

**Fig 6:**
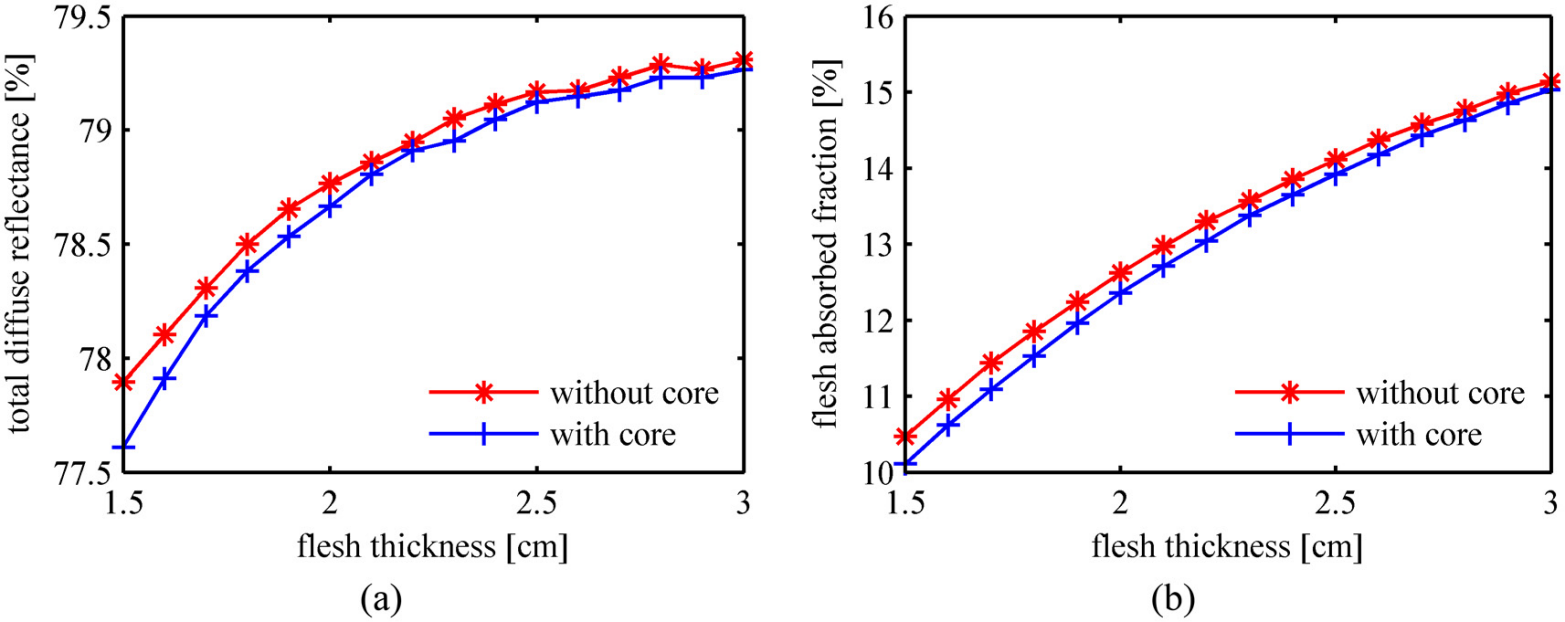
Light transport features in peaches with and without fruit core. (a) Total diffuse reflectance; (b) Flesh absorbed fraction.

The MC-simulated total diffuse reflectance and the flesh absorbed fraction versus the flesh layer thickness are plotted in Fig 6a and 6b, respectively. Differences are found between the two curves representing the simulation results with and without the fruit core. Comparison between the calculated profiles confirms the theoretical analysis. The effects of the fruit core in the stone fruit should be considered to obtain accurate optical transport features. The skin-flesh-core models for the stone fruit were used in the following simulations.

### Effects of the fruit skin

Peaches are thin-skinned fruits with a skin thickness *d*_1_ varying in a range of 0.01 to 0.05 cm for most peaches. The optical properties listed in Table 2 were used in the MC simulations whereas the thickness of the peach flesh layer was fixed at *d*_2_ = 1.5 cm.

**Fig 7:**
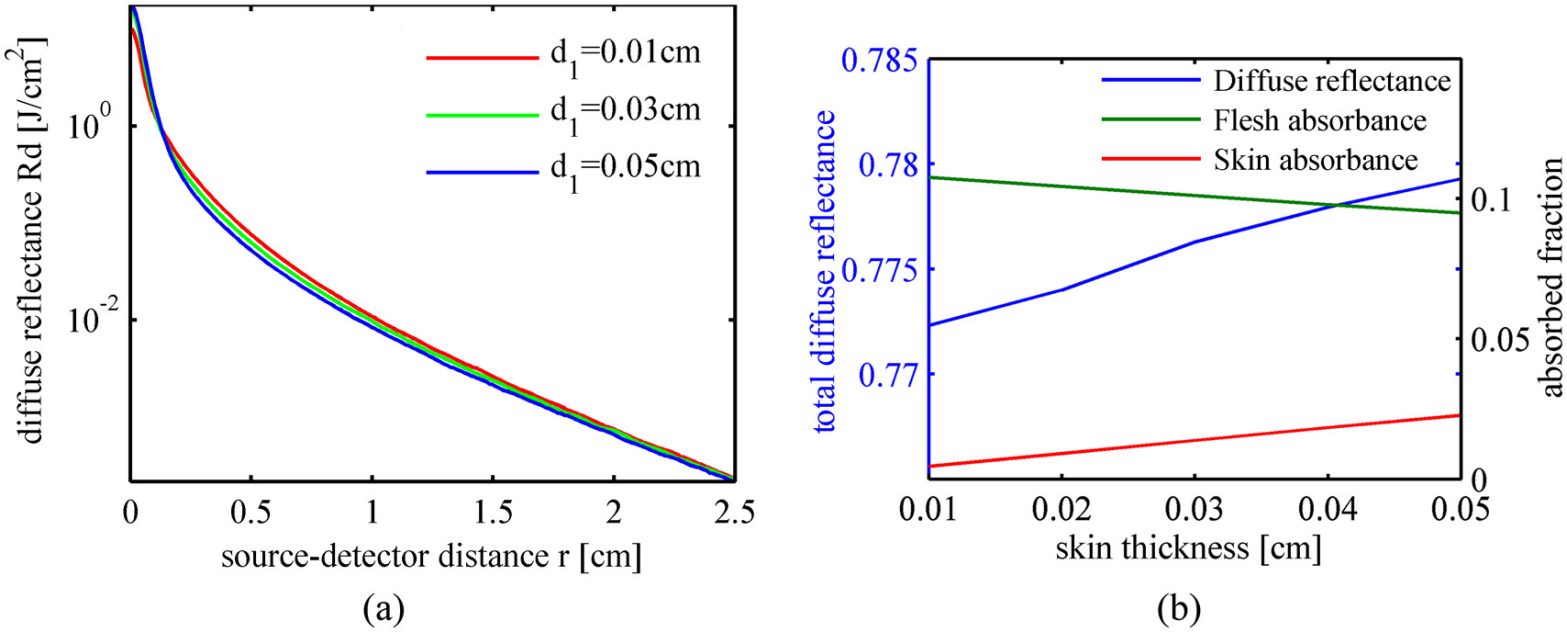
Light transport features in peaches with different skin thicknesses. (a) Spatially resolved diffuse reflectance; (b) Total diffuse reflectance, flesh absorbed fraction and skin absorbed fraction.

In the discussion of light scattering in food, Birth confirmed that the light scattering elements in plant leaves were the cell walls [23]. McGlone reported that it was probably the same with fruit, and the cell wall interfaces would be the dominant light scatterers [24]. There are large, fluid filled sacs in the peach flesh tissue. This suggests that the scattering coefficient is lower in the peach flesh tissue than in the skin tissue. In our experiments, peach skin tissue was more than three times more scattering than the flesh tissue (*μ*_*s*1_ = 102 cm^-1^ and *μ*_*s*2_ = 28.4 cm^-1^). The highly scattering skin tissue has two influences on the spatially resolved diffuse reflectance. First, in the region closest to the incident point (~ *r* < 0.15 cm), more photons are back scattered into the air by the highly scattering skin tissue and are detected as diffuse reflectance. Therefore, the spatially resolved diffuse reflectance increases with the skin thickness as shown in Fig 7a. Second, fewer photons will reach the flesh tissue when the skin becomes thicker, and the highly scattering skin tissue reduces the proportion of these photons propagating back into the air through the skin tissue. In the region of *r* within 0.15 ~ 1.5 cm, diffuse reflectance decreases with an increase in the skin thickness. In the further region (~ *r* > 1.5 cm), the effects of the peach skin on the diffuse reflectance are not significant. Fig 7b gives the total diffuse reflectance, flesh absorptance and skin absorptance versus the skin thickness. If the detector is positioned near the incident point (~ *r* < 1.5 cm in our simulations) or if the fruit to be measured is thick-skinned, the effects of the skin tissue should be taken into consideration.

**Fig 8:**
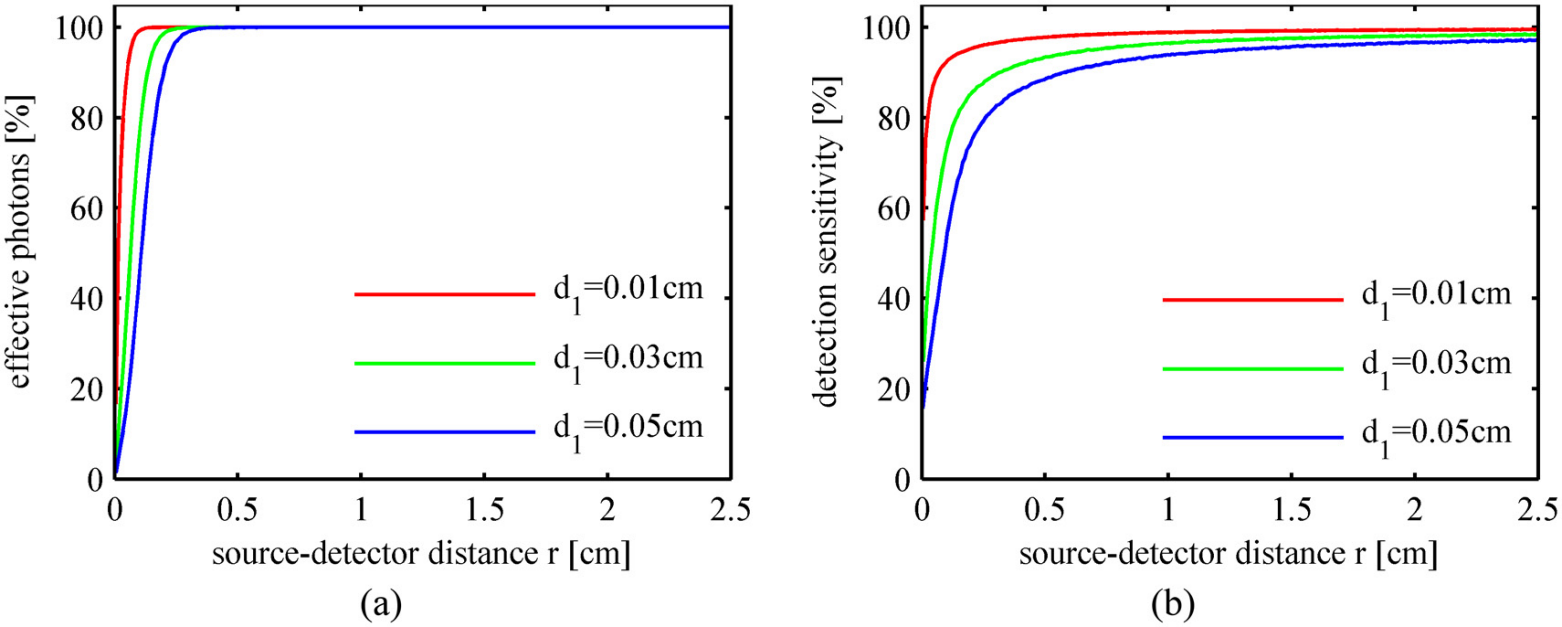
Detection efficiency for peaches with different skin thicknesses. (a) Percentage of the effective photons; (b) Detection sensitivity of the flesh tissue.

The percentages of the effective photons in the diffuse reflected light and the detection sensitivities of the flesh tissue for the peach fruit with different skin thicknesses are plotted in Fig 8a and 8b, respectively. It is reasonable that the detection efficiency decreases when the skin thickness increases especially in the region close to the incident point. To obtain sufficient detection efficiency, the detection position should not be too close to the incident point.

### Effects of the fruit flesh

**Fig 9:**
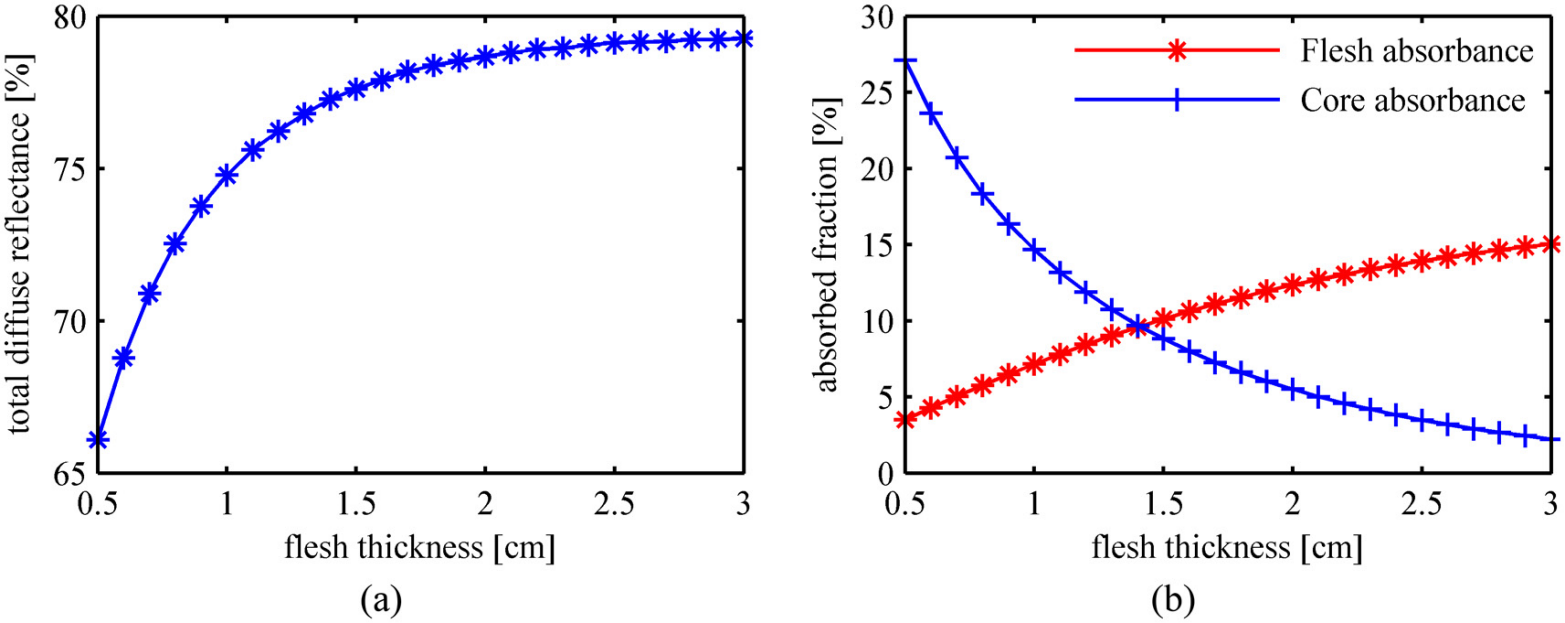
Light transport features in peaches with different flesh thicknesses. (a) Total diffuse reflectance; (b) Absorbed fraction by the flesh and the core.

**Fig 10:**
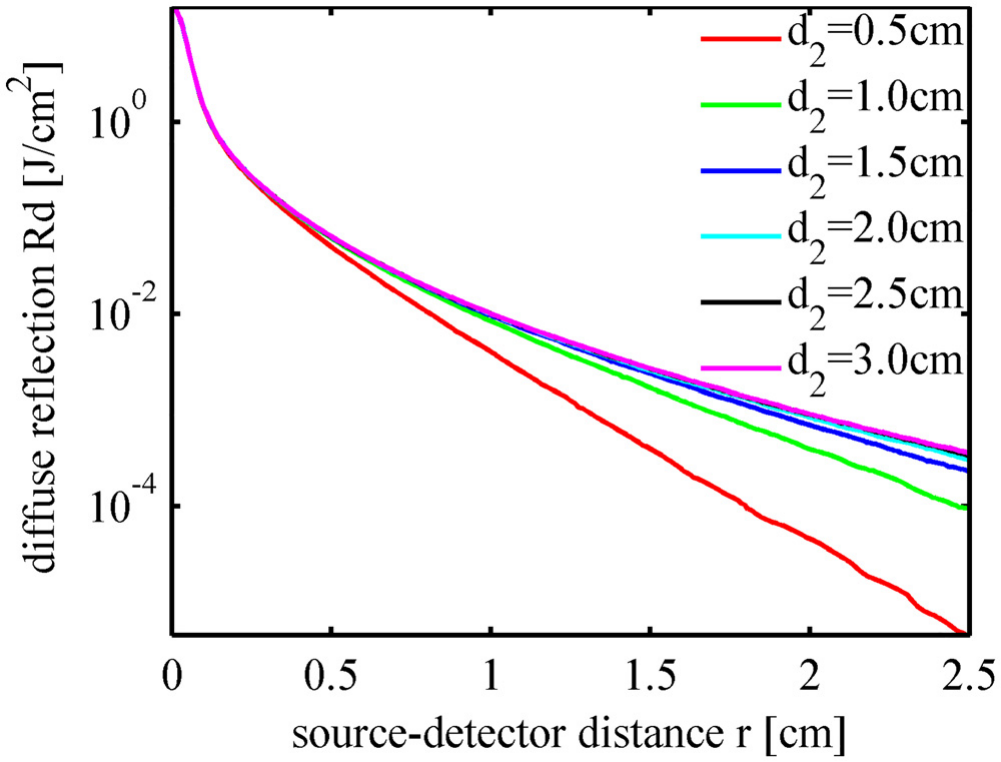
Spatially resolved diffuse reflectance for the peaches with different flesh thicknesses.

**Fig 11:**
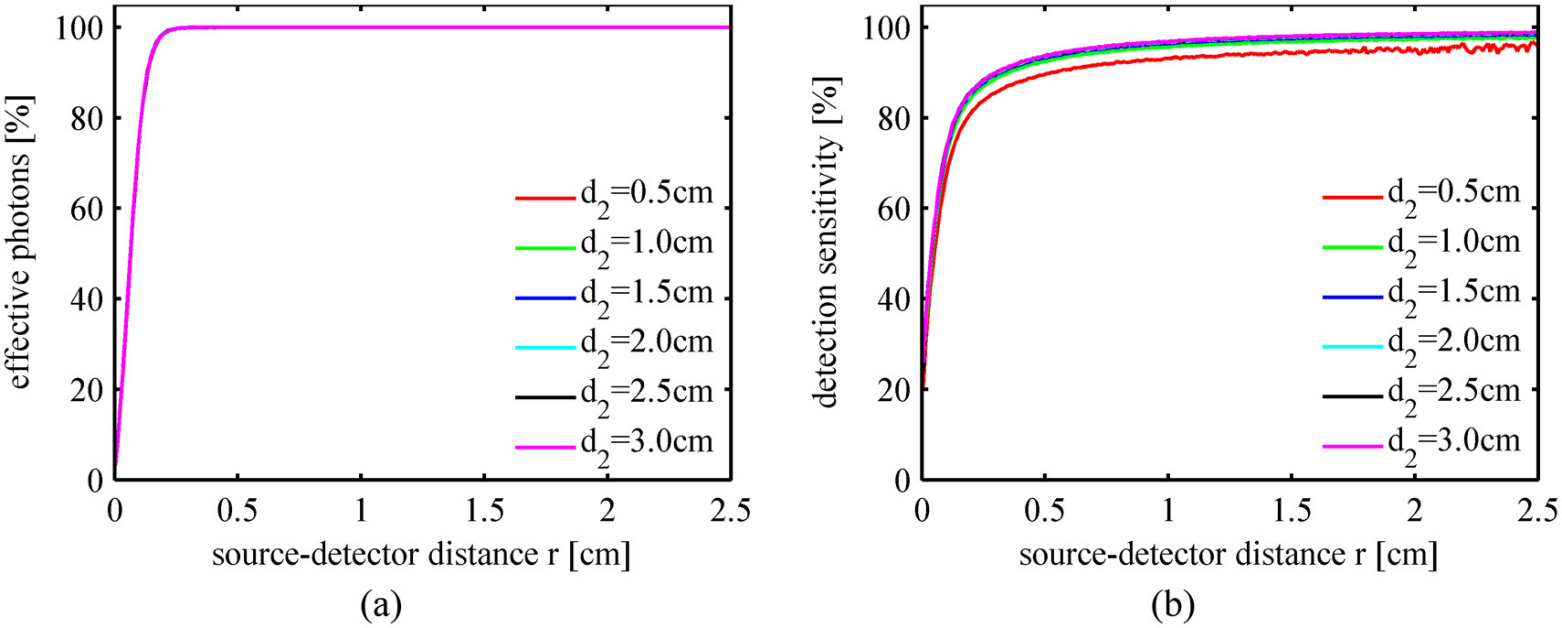
Detection efficiency with different flesh thicknesses. (a) Percentage of effective photons; (b) Detection sensitivity of the flesh tissue.

The fruit flesh is the target layer in fruit quality inspection. We simulated light propagation in peach tissues using the parameters listed in Table 2 except for varying the flesh thickness in the range of 0.5 ~ 3.0 cm. The simulation results in Fig 9 indicate that the total diffuse reflectance and the flesh absorption rate tend to increase slightly with the thickness of the flesh layer, whereas the fruit core will absorb less intensity when the flesh layer becomes thicker. Most (if not all) peach fruits have a flesh layer with a thickness of 1.5 ~ 3.0 cm. Fig 10 indicates that there are no significant differences between the spatial profiles of the diffuse reflectance for peaches with the same optical properties. Additionally, Fig 11 indicates that the detection efficiency is not sensitive to flesh thickness as well. However, as seen in Fig 10, the diffuse reflectance decreases obviously with the flesh thickness in the range of 0.5 ~ 1.5 cm. Simulation results suggest that, in the inspection of small stone fruits, such as cherries, the influence of the flesh thickness should be taken into consideration.

**Fig 12:**
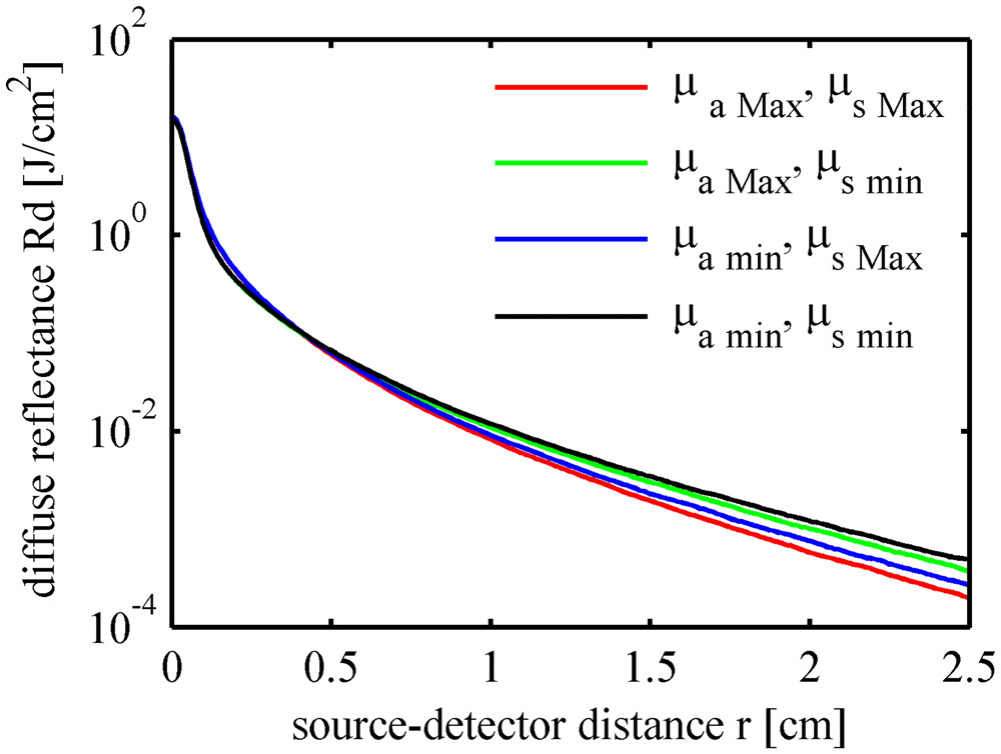
Spatially resolved diffuse reflectance in the fruit tissues with different optical properties.

**Fig 13:**
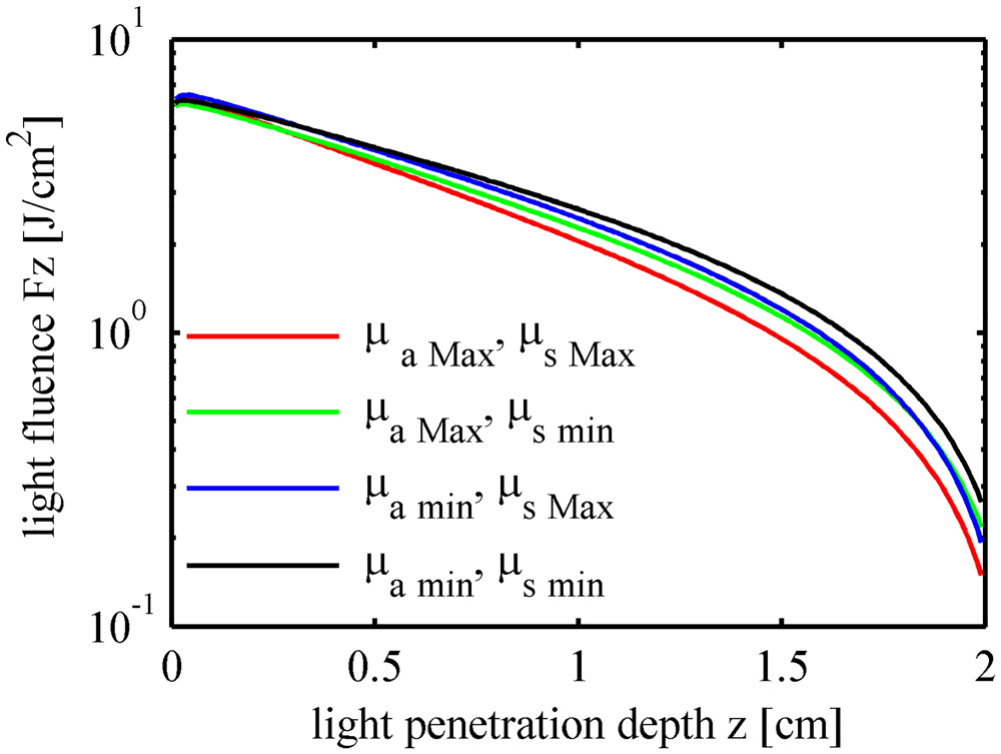
Light fluence in the fruit tissues with different optical properties.

The optical properties of the fruits are characterized by the absorption coefficient *μ*_*a*_ and reduced scattering coefficient *μ’*_*s*_ = (1-*g*) *μ*_*s*_. To further evaluate the influence of *μ*_*a*_ and *μ’*_*s*_, the values of *μ*_*a*_ and *μ*_*s*_ of the flesh tissue were altered within a ±20% range relative to the expected mean values given in Table 2, whereas the thicknesses of the skin and the flesh layers were fixed at 0.03 cm and 2.0 cm, respectively. The patterns of the diffuse reflectance (Fig 12) and light fluence in fruit tissues (Fig 13) were calculated using four pairs of *μ*_*a*_ and *μ*_*s*_ including the minima and the maxima. Differences were found between the four curves. Fruit tissue with a large *μ*_*a*_ value absorbs light energy rapidly in short distances, whereas light in the tissue with a small *μ’*_*s*_ tends to propagate forward to the deeper areas of the sample. Table 3 lists the total diffuse reflectance, the flesh absorption rates, and the core absorption rates. The simulation results show that up to 14.47% of the photons were absorbed by flesh tissue under the maximum absorption condition (*μ*_*a*,Max_, *μ*_*s*,min_). The flesh absorption rate reduced to 13.72% when *μ*_*s*_ increased from the minimum to the maximum whereas *μ*_*a*_ remained constant as the maximum (*μ*_*a*,Max_, *μ*_*s*,Max_). Up to 81.41% of the photons exit as diffuse reflectance under the maximum scattering condition (*μ*_*a*,min_, *μ*_*s*,Max_).

**Table 3:** Light transport features in the fruit tissues with different optical properties.

*μ*_*a*_ and *μ*_*s*_ of flesh (cm^-1^)	Total diffuse reflectance	Flesh absorbed fraction	Core absorbed fraction
*μ*_*a*,mean_ = 0.024, *μ*_*s*,mean_ = 28.4	0.7867	0.1236	0.0550
*μ*_*a*,Max_ = 0.029, *μ*_*s*,Max_ = 34.1	0.7901	0.1372	0.0379
*μ*_*a*,Max_ = 0.029, *μ*_*s*,min_ = 22.7	0.7550	0.1447	0.0661
*μ*_*a*,min_ = 0.019, *μ*_*s*,Max_ = 34.1	0.8141	0.1018	0.0490
*μ*_*a*,min_ = 0.019, *μ*_*s*,min_ = 22.7	0.7799	0.1053	0.0801

The light transport features in fruits are determined primarily by the absorption and reduced scattering coefficients of the flesh tissue. Research into the correlation between the optical properties and the physical-chemical and mechanical parameters of fruits have suggested that the absorption coefficient at certain wavelengths could provide information about the content of sugar, water, chlorophyll, etc., and the scattering coefficient could be used to estimate firmness [25, 26]. The optical properties of the fruit tissue can be determined from the vis-NIR spectroscopy measurements, and the internal structure and quality can be inversely deduced.

### Discussions on the measurement schemes

#### Choice of source-detector distance

The properties of the fruit flesh tissue are of highest concern during fruit quality inspection. It is important to increase the optical pathlength of the photons in flesh tissue, to improve the detection sensitivity, and to depress the influence of the skin and the core. As shown in Figs 8 and 11, the detection efficiency increases with the radial distance, i.e., the source-detector distance in interactance measurement mode. The detection position should not be too close to the incident point especially when the fruit is thick-skinned. However, the radially resolved diffuse reflectance decreases exponentially with the radial distance, as shown in Figs 7, 10 and 12. High incident intensity and a detector with a high sensitivity are required if the source-detector distance is large.

#### Choice of detection angle

**Fig 14:**
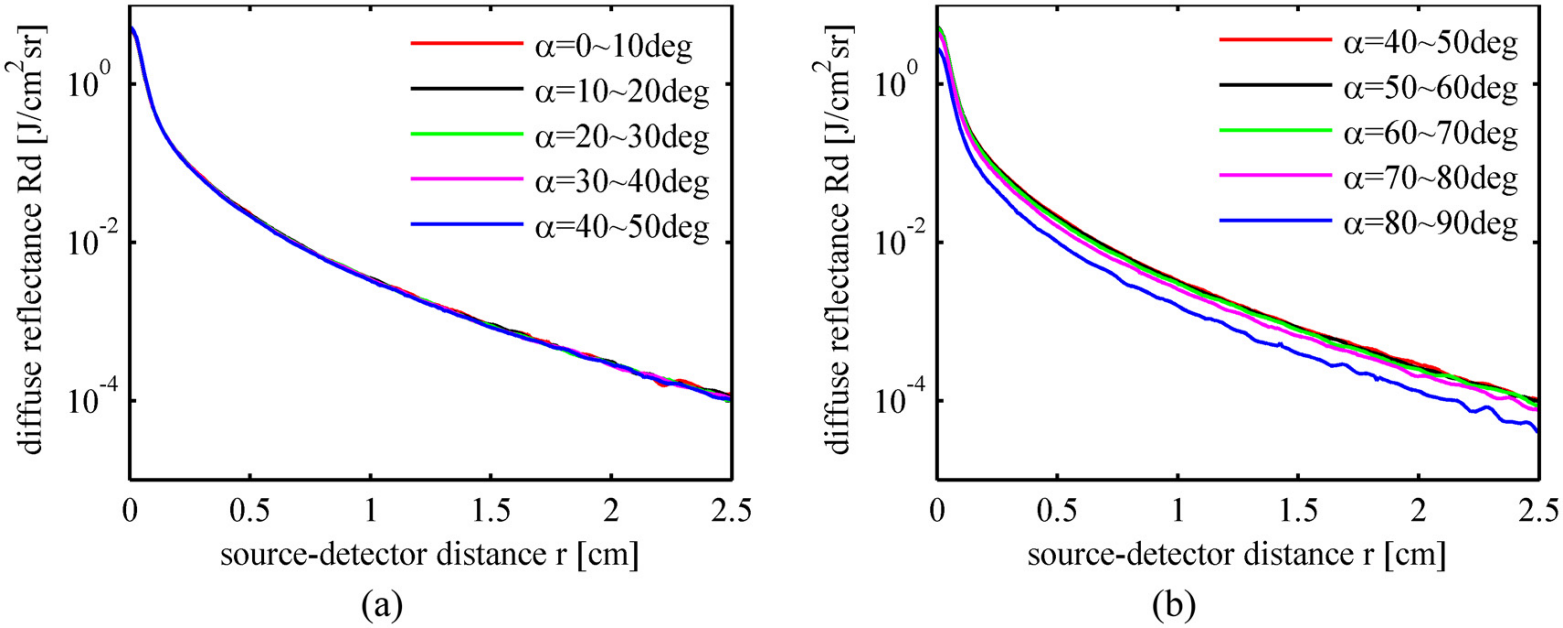
Diffuse reflectance versus radial distance per solid angle. (a) Detection angles range from 0~50 degrees at 10 degrees intervals; (b) Detection angles range from 40~90 degrees at 10 degrees intervals.

The diffuse reflected photons escape into the air from different directions. The choice of light detection angle may influence the sensitivity and accuracy of the diffuse reflectance spectral measurement. An MC simulation was applied to calculate the photon probability per unit area perpendicular to the photon direction per solid angle. The expected mean values of the optical properties in Table 2 were used in the simulation. A convolution was taken to obtain the response for a Gaussian incident light beam. The averaged diffuse reflected energy densities per unit area per solid angle are plotted in Fig 14. There was no significant difference in the angular resolved diffuse reflectance when the detection angle changes in the range of 0~80 degrees. However, the probability for photons to exit the fruit tissue surface at exiting angles of 80~90 degrees is much less. The results indicate that a large detection angle near 80~90 degrees should be avoided to improve the detection efficiency and sensitivity of diffuse reflectance spectral measurement.

#### Choice of source intensity

A sufficient number of photons must be collected by the detector, whereas high incident light intensity can easily burn the fruit surface. In addition to the optical properties of the fruit, the source-detector distance, the total acceptance angle and area, the detection direction, and the sensitivity of the detector have to be taken into account. Accurate MC simulations can aid in selecting a source intensity for a given measurement system to achieve the optimal sensing results.

## Summary and Conclusions

MC simulations were performed to follow each photon of the incident light beam inside the peach fruit tissues. Although we are primarily interested in the properties of the flesh, photon propagation in the stone fruit is influenced by the skin and core properties. Based on the simulation results, proper modeling of light transport through intact stone fruit should be performed using a model that consists of skin, flesh, and core layers.

The effects of the optical properties of the fruit flesh on the patterns of diffuse reflectance, light fluence and absorption were analyzed. The optical properties of the fruit tissue can be retrieved from the vis-NIR spectroscopy measurements, and the internal structure and quality can be inversely deduced based on the correlation between the optical properties and the physical-chemical and mechanical parameters of the fruits.

The detection efficiency was evaluated by the percentage of effective photons and the detection sensitivity of the flesh tissue. The detection efficiency is low if the detector is placed too close to the incident point, whereas a high incident intensity and a detector with high sensitivity are required if the source-detector distance is large. The choice of detection angle was discussed by comparing the angular resolved diffuse reflectance. A large detection angle should be avoided to improve the detection efficiency and sensitivity of the diffuse reflectance spectral measurement. When used with broadband light and a spectrometer, the development of reliable MC simulation tools could also allow computation of optimal configurations without the need for extensive experimental measurements. The analysis can aid in the design of a more effective measurement configuration (e.g., source intensity, wavelength range, detector position and sensing area).

Research is presently in progress to simulate light transport features in fruit tissues with complex shapes rather than multi-layered models.

## Abbreviations

vis-NIR: visible-near infrared
RTE: radiation transport equation
MC: Monte Carlo
IAD: inverse adding-doubling
NIR: near infrared
